# Are Black Phosphorus Hydrogels Antimicrobial Without Photonic Activation?

**DOI:** 10.3390/molecules30112292

**Published:** 2025-05-23

**Authors:** Leon D. Pope, Shreehari Kodakkat, Aaron Elbourne, Peter C. Sherrell, Nhiem Tran, Kate Fox

**Affiliations:** 1 School of Engineering, RMIT University, 124 La Trobe St., Melbourne, VIC 3000, Australia; leon.pope@rmit.edu.au; 2 School of Science, RMIT University, 124 La Trobe St., Melbourne, VIC 3000, Australia; s4010118@student.rmit.edu.au (S.K.); aaron.elbourne@rmit.edu.au (A.E.); peter.sherrell@rmit.edu.au (P.C.S.)

**Keywords:** black phosphorus, hydrogel, antibacterial, bacteria, antibiotic resistance

## Abstract

Black phosphorus (BP) nanoflakes have attracted interest as an antimicrobial material for wound healing and implant-associated infections due to their bactericidal activity without the use of antibiotics. Hydrogels are frequently used as a delivery system; however, most research uses photonic activation in the form of near-infrared (NIR) laser stimulation to cause rapid BP degradation, reactive oxygen species (ROS) generation, and a localized photothermal effect. For implant-coating applications, using NIR laser stimulation could be challenging in practice, especially for porous orthopedic implants. This article investigates whether BP nanoflakes, suspended in Pluronic F127 (F127) hydrogels, remain effective against Staphylococcus aureus without photonic activation. The experimental results showed 89.4 ± 7.6% bacterial inhibition from BP nanoflakes at a 5120 µg/mL concentration via passive diffusion in F127; however, it could not kill all the bacteria present. It is hypothesized that the F127 gel interface could create a barrier between the bacteria, which continue to multiply in media, and the antimicrobial black phosphorus compound, which degrades in the F127.

## 1. Introduction

Bacterial infection and biofilm formation are major causes of failure for all implantable medical devices [1]. Bacterial biofilms are caused by an accumulation of bacteria that are attached to the implant. These bacteria are encased in a film of an extracellular polysaccharide matrix secreted by the bacteria. Biofilms present key challenges to patient outcomes post-surgery, as they can be 10–1000 times more resistant to the effects of antimicrobial agents, making chronic and recurrent infections more prevalent [2] and contributing to antibiotic resistance. In addition to putting severe economic strain on healthcare systems, chronic infections greatly increase patient morbidity and mortality rates [3].

Recently, black phosphorous (BP) nanoflakes have been investigated for their broad-spectrum antimicrobial properties [4]. BP is a 2D allotrope of phosphorus and exhibits antimicrobial properties through various mechanisms, including direct physical contact-driven cell membrane damage, photothermal activity, and reactive oxygen species (ROS) production [4]. This non-antibiotic-reliant mechanism means that BP may be a part of a future toolbox to address so-called “superbugs” (antibiotic-resistant bacteria) [4]. BP nanoflakes with lateral sizes ranging from 200 nm to 5 μm have been shown to have antimicrobial and antifungal properties across a wide range of bacteria and fungi that can cause biofilms on medical implants [4,5]. Shaw et al. [5] demonstrated that after two hours of direct physical contact with ~900 ng/cm^2^ BP nanoflakes on glass Petri dishes, the average proportion of nonviable methicillin-resistant *Staphylococcus aureus* (MRSA) and drug-resistant pathogenic fungi *Cryptococcus neoformans* was 97.9% and 99.3%, respectively. Moreover, antimicrobial activity occurred in dark conditions without photonic activation, suggesting contact-driven membrane damage from the morphological properties of the 2D nanoflakes, which penetrate microbe cell membranes or singlet oxygen (^1^O_2_) and superoxide radical (O_2_^•−^) generation to be the active antimicrobial mechanisms.

In another study, Sun et al. [6] exposed Gram-negative *Escherichia coli* and Gram-positive *Staphylococcus aureus (S. aureus)* to liquid-exfoliated BP nanoflakes at concentrations ranging from 80 to 1280 µg/mL to study their antibacterial efficacy. The minimum inhibitory concentration (MIC) of liquid-exfoliated BP nanoflakes was determined to be 160 µg/mL for both bacteria species. However, in this study, the antimicrobial effects were enhanced by BP’s photoactivity. The BP–bacteria solution was irradiated by an 808 nm NIR laser for 3 min before being cultured on an agar plate for 16 h. It was found that exposing the BP nanoflakes to a light source in the visible to near-infrared (NIR) region produced singlet oxygen (^1^O_2_) ROS and a photothermal effect, both of which are detrimental to the survival of the bacteria [4,6].

To apply the antimicrobial properties of BP nanoflakes to clinically relevant causes, hydrogels have been investigated as a potential delivery system. Depending on whether the intended use is as a topical antimicrobial or as an implant coating, a variety of gelatin methacrylate [7,8,9], chitosan [10,11,12], and hyaluronic acid [13,14,15]-based hydrogels have been investigated. However, in all cases, photonic activation is used to trigger rapid ROS generation and the photothermal effect of BP nanoflakes to provide high bactericidal performance.

Critically, while the emerging literature has shown the efficacy of BP nanoflakes in killing these bacteria and fungi [4,5], there has been minimal study on the efficacy of the integration of antibacterial BPs into hydrogels without photonic activation. If BP hydrogels are effective antimicrobial agents without photonic activation, they could be applied to medical implants, providing local antimicrobial activity without the use of antibiotics and without triggering cytotoxic environments from excessive heat that can induce severe inflammation and damage to adjacent tissues.

Herein, we provide a study of the bactericidal activity of BP nanoflakes suspended in hydrogels of F127 without photonic activation. F127 was chosen as a hydrogel matrix as it has been investigated as a drug delivery system to deliver antibiotics for the local prophylaxis of infection since the mid-1990s [16,17,18]. F127 is a triblock copolymer consisting of polyethylene oxide (PEO)-polypropylene oxide (PPO)-polyethylene oxide (PEO) and can form semisolid gels at body temperatures, depending on the concentration [19]. Below their gelation temperature, F127 solutions do not exhibit structural ordering; however, as temperature increases, the hydrophobicity of PPO increases, creating micelles consisting of PPO cores surrounded by a PEO shell [20]. Above F127’s gelation temperature, micelles are packed into a cubic lattice with entanglements between PEO chains in overlapping micelles [20]. During drug or nanoparticle delivery, the cubic matrices are sheared apart from gradual gel dissolution into their surrounding aqueous environment [19,21]. F127 has also been used as a drug delivery device to provide osteoconductive compounds in 3D-printed titanium scaffolds [22,23], making it especially translatable for the application of 3D-printed implant surface treatment. Additionally, the antibiotic vancomycin has been loaded into F127 as a control, as it is effective against a wide range of Gram-positive bacteria, including *S. aureus*, which are the most prevalent pathogens that cause orthopedic prosthetic infections [24].

This work provides guidance for the future design of BP-laden hydrogels as antimicrobial agents without the use of illumination to enhance BP degradation.

## 2. Results and Discussion

### 2.1. Black Phosphorus Characterization

**Figure 1:**
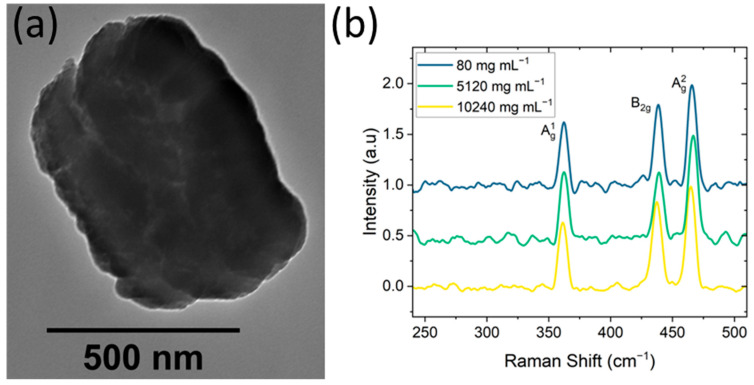
BP nanoflake characterization. (**a**) TEM micrograph of typical black phosphorus nanoflakes. Lateral sizes ranging from 500 to 1000 nm. (**b**) Raman spectra of 80, 5120, and 10,240 µg/mL concentrations of dried exfoliated BP nanoflakes deposited on a silicon wafer.

Commercially available BP powder was sonochemically exfoliated in Milli-Q water, producing nanoflakes with an average lateral size of 700 nm by transmission electron microscopy (TEM) (Figure 1a) and dynamic light scattering (679 ± 230 nm, Figure S1). The BP was shown to remain intact post-exfoliation at all studied concentrations (from 80 µg mL^−1^ to 10,240 µg mL^−1^) via Raman spectroscopy (Figure 1b). Across all nine concentrations assessed, distinct peaks corresponding to black phosphorus were identified: A1g (361 cm^−1^), B2g (438 cm^−1^), and A2g (465 cm^−1^) [24]. BP nanoflakes with similar dimensions have been shown to provide antimicrobial properties in in vitro and in vivo murine animal models when exposed to NIR and ambient light conditions [6,25].

### 2.2. BP F127 Gel Characterization

**Figure 2:**
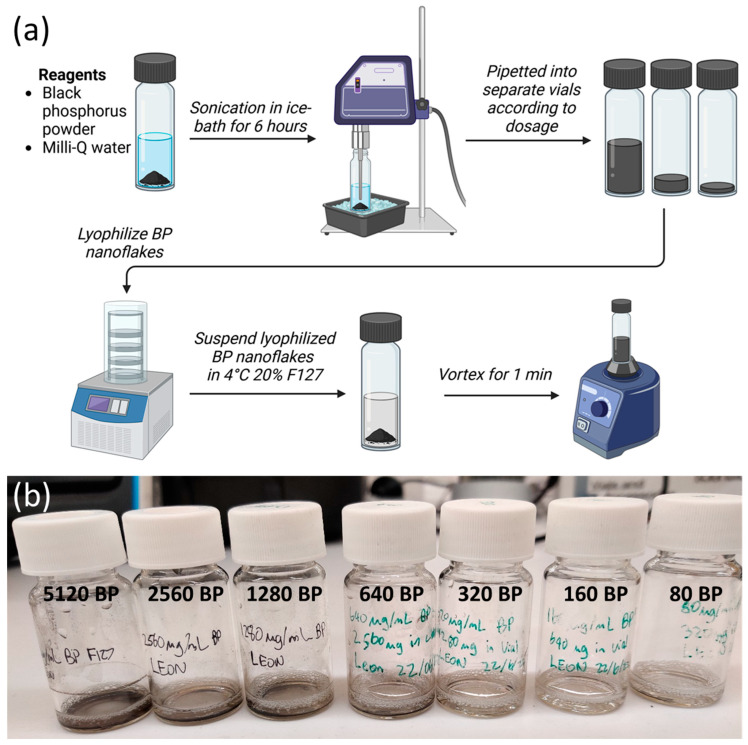
BP F127 preparation. (**a**) BP F127 preparation flowchart. (**b**) Photo, from right to left, of 80, 160, 320, 640, 1280, 2560, and 5120 µg mL^−1^ BP F127, showing a change in opacity across the range of concentrations tested.

The produced BP nanoflakes were then pipetted into separate vials according to the concentrations tested and lyophilized to prevent oxidation. The 20% *w*/*v* F127 in its liquid state at 4 °C was then added to the lyophilized BP nanoflakes, followed by vortex mixing to disperse the BP throughout the F127 gel (Figure 2). At body temperature (37 °C), sol–gel transition occurs, creating a gel matrix that can impede BP nanoflake dispersion.

**Figure 3:**
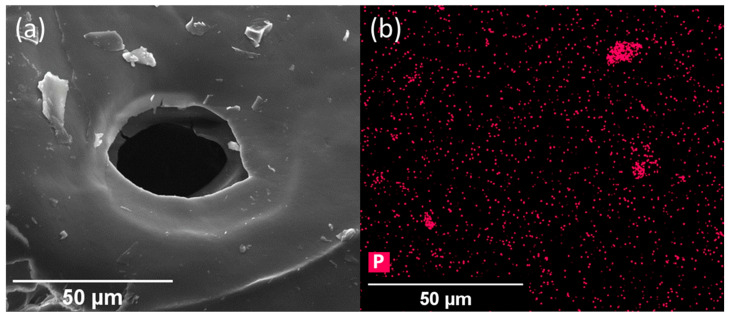
A demonstration of the dispersion of BP in F127. (**a**) An SEM micrograph of freeze-dried 2560 µg mL^−1^ BP F127 hydrogel showing a microporous environment from sublimation. (**b**) An EDS map of (**a**) showing characteristic X-rays of phosphorus (P) within the hydrogel.

To characterize the BP F127 gels produced, the samples underwent lyophilization and were examined via scanning electron microscopy (SEM) and energy-dispersive X-ray spectroscopy (EDS), showing phosphorus characteristic X-ray signals within the hydrogel (Figure 3a,b and Figure S2).

Before lyophilization, the BP F127 samples were aggressively vortexed until visually homogenous; then, they were rapidly frozen. After lyophilization, BP F127 samples contained phosphorus-characteristic X-rays throughout the hydrogel matrix; however, individual BP nanoflakes were difficult to identify on the lyophilized hydrogel substrate. Microscale BP flakes were more easily identified and were distributed throughout the hydrogel matrix. These results show that BP was successfully integrated into F127 hydrogels at sufficient concentrations to test their efficacy as antimicrobial agents.

### 2.3. F127 Dissolution

As the presence of F127 gel influences the release of BP nanoflakes into media, which presumably affects their antibacterial activity, an F127 dissolution experiment was conducted. For this, the dissolution of 50 µL F127 in 200 µL of phosphate-buffered saline (PBS) solution was examined. This was performed by incubating the F127 in PBS at 37 °C and measuring the weight change in F127 over time. The result shows that without stirring, 53.3% F127 dissolution was observed in the first 6 h, and 73.3% dissolution was observed after 24 h (Figure S3). This result suggests a burst release of BP nanoflakes from F127 gels occurring during this time window. However, some released flakes may remain coated in F127, which can alter their potential antimicrobial performance. Recent work has shown that F127 can create micellular-like coatings on the BP, enabling its continued protection from oxidation as BP is encapsulated in the hydrophobic PPO core [26]. This can reduce the oxidation rate and, therefore, ROS production, which is the primary antimicrobial mechanism of BP nanoflakes without photonic activation. However, F127 micelles have an average spherical radius of 4.4 nm, which is independent of concentration and temperature [27]. As the average size of BP nanoflakes was 679 ± 230 nm, as identified by dynamic light scattering (Figure S1), the F127 encapsulation of BP nanoflakes is unlikely to impede ROS release into solution.

### 2.4. In Vitro Antibacterial Activity of BP F127 Gels

**Figure 4:**
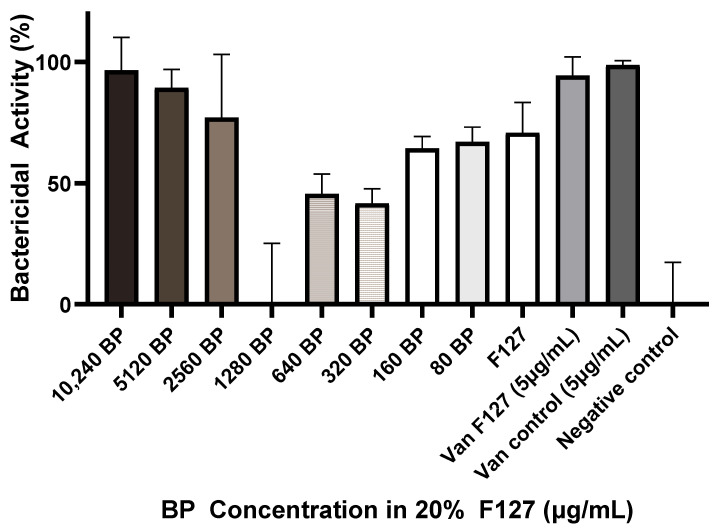
Bactericidal activity of BP and VAN F127 gels, measuring the OD600 absorbance of *S. aureus* cultured for 24 h when exposed to the gels.

**Figure 5:**
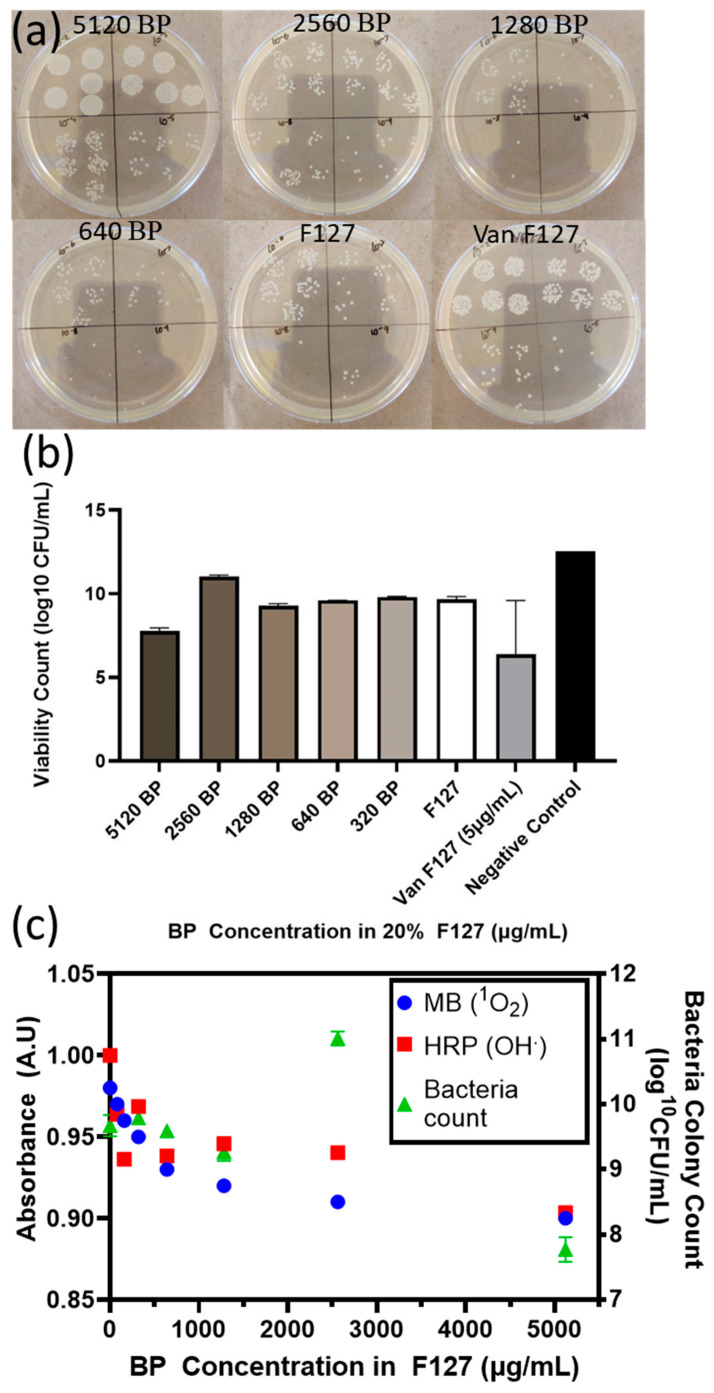
CFU/mL counts of *S. aureus* exposed to BP and Van-loaded 20% *w*/*v* F127 gels. (**a**) Serial dilutions of media from each BP and Van F127 well. (10^−2^–10^−9^) onto TSB agar plates. (**b**) Tabulated data from counting colonies of *S. aureus* exposed to BP and Van F127 gels for 24 h. (**c**) Correlating CFU/mL count sto ROS radicals from BP F127 after 48 h.

The in vitro antibacterial activity of the BP F127 gels was measured using concentrations between 10,240 and 80 µg/mL. Vancomycin (Van) and Van-loaded F127 gels were tested as positive control samples, and 50 µL of MilliQ water was used as a negative control. By measuring the optical density of *S. aureus*-inoculated media exposed to BP F127 gels for 24 h, it was alluded that bactericidal activity occurred at concentrations > 5120 µg/mL (Figure 4), showing 89.4 ± 7.6% bactericidal activity for 5120 µg/mL and 97.6% ± 13.6% for 10,240 µg/mL BP F127. This active antibacterial mechanism is likely attributed to ROS generation as BP nanoflakes encapsulated in F127 hydrogels would not have significant direct contact with bacteria. To determine ROS generation from BP F127 gels, singlet oxygen species (^1^O_2_), hydroxide (OH^•^), and superoxide (O_2_**^•^**^−^) radicals were screened for using methylene blue (MB), horseradish peroxidase (HRP) enzyme, and xanthine oxidase (XO) (Figure S4 and Figure 5c). F127, on its alone, also inhibited bacteria activity, with a 70.8 ± 12.5% reduction compared to the negative control. While F127 does not have inherent bactericidal properties, the reduction in bacterial activity could be attributed to F127 causing a decreased adhesion of fibronectin between the hydrogel and the extracellular matrix of *S. aureus*, limiting protein adsorption compared to plastic tissue cultures [28].

It was also noted that sub-lethal doses may have caused a stimulatory effect, with 1280–80 BP µg/mL concentrations having less bacterial inhibition compared to F127 gel without any antimicrobial compounds. This stimulatory effect is a cause for concern in the future design of antimicrobial BP agents, as it suggests that low concentrations of BP that passively degrade may lead to the formation of biofilms rather than their prevention. This may be attributed to the low ROS production rate generated by degrading BP in dark conditions, as previous studies have observed that sub-lethal superoxide can facilitate bacteria growth and colonization [29]. Additionally, *S. aureus* exposed to sub-lethal ROS by macrophages has been shown to cause antibiotic tolerance [30]; thus, if low-dose BP without photoactive stimulation is compounded with antibiotics, antibiotic resistance may be expedited.

Serial dilutions from each well were used to calculate the amount of CFU obtained after exposure to the BP F127 gels (Figure 5). The results from serial dilutions confirmed a large number of bacteria within all samples, with all BP samples having too many colonies to count at a dilution factor of 10^4^.

The bactericidal effect of BP F127 was most visible at a 5120 µg/mL BP concentration, with the CFU count reduced to 10^7.7^ CFU /mL compared to the 10^9.7^ CFU/mL negative control, representing a two-log reduction in CFU. However, the bactericidal activity was not strong enough to completely prevent bacterial proliferation. The BP nanoflake concentrations assessed were significantly higher than concentrations tested from the previous literature, which reported positive bactericidal results, as shown in Table S1. In cases where BP nanoflakes were bactericidal inside a hydrogel, an NIR laser or light stimulation was used to stimulate rapid ROS generation and a local photothermal effect. Without NIR laser or ambient light irradiation, the BP nanoflakes encapsulated in F127 did not degrade rapidly enough in dark conditions to provide adequate ROS generation and lethal oxidative stress. From the ROS screening results (Figure S4), no significant ROS generation was identified after incubating BP F127 gels for 24 h. After 48 h, singlet oxygen species and hydroxide radicals were identified, but no superoxide radicals were present. In cases where BP nanoflakes were bactericidal in dark conditions, the bacteria were in direct contact with BP nanoflakes or in water, where the rapid oxidation of BP can occur to disrupt cell membranes [5,31]. This, therefore, suggests that without photonic activation, F127 prevents BP nanoflakes from rapidly degrading, limiting the ROS generation rate, or the hydrogel interface creates an insulated barrier between the degrading BP nanoflakes and the proliferating bacteria.

**Figure 6:**
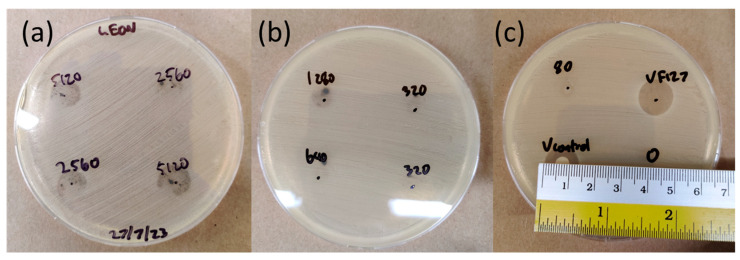
Disk diffusion test of BP nanoflakes and VAN F127 on *S. aureus*-inoculated MH agar. (**a**) Zones of inhibition suggested antibacterial activity at 5120 µg/mL and milder activity at 2560 µg/mL; (**b**) No zones of inhibition recorded from BP nanoflake F127 samples with 1280 µg/mL and below. (**c**) Measuring zone of inhibition of vancomycin control in Milli-Q (using 10 µL droplet of 500 µg/mL on filter paper).

To further study the antibacterial activity of BP nanoflakes in F127 gel, a disk diffusion experiment was conducted in which 10 µL of BP gels was pipetted directly onto an inoculated Mueller–Hinton (MH) agar plate and incubated in dark conditions (Figure 6). At the highest tested BP concentration of 5120 µg/mL, the inhibition of *S. aureus* growth was observed. However, the antibacterial activity was restricted within the area covered by the gel droplet, suggesting that either BP did not diffuse out of the gel droplet or the amount of diffused BP was not sufficient in inhibiting *S. aureus* growth. Mild antibacterial activity was observed at a BP concentration of 2560 µg/mL, with bacterial growth visible in the gel droplet; however, this was not as vigorous as in areas without the gel. All other BP F127 concentrations showed a bacterial lawn in the area of the hydrogel droplets, indicating no observable antibacterial activity.

**Figure 7:**
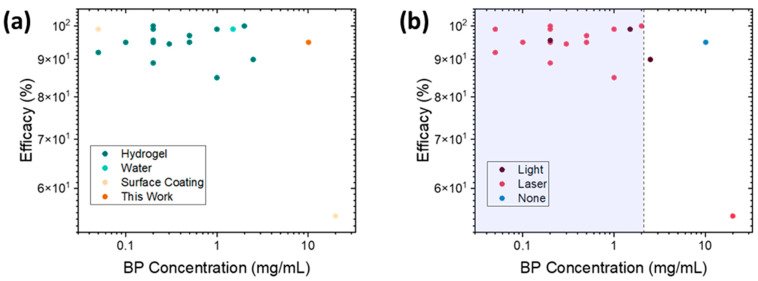
A comparison of reported antimicrobial BP in the literature; (**a**) how the BP was delivered or used; (**b**) how antimicrobial activity was activated, using a laser (typically an 808 nm NIR laser), light (ambient light or a Xenon lamp), or passive activity in dark conditions. The shaded area represents concentrations where work bacteria stimulation (rather than bactericide) occurred in an equivalent passive release system in this work. References and expanded details are provided in Table S1.

These results highlight a key challenge in the use of BP for antimicrobial applications. If presented as an implant coating strategy where the BP is encapsulated in a hydrogel that coats an implant at commonly studied loadings (summarized in Table S1), the BP may, in fact, have a stimulative effect on bacterial growth (Figure 4) if there is no photonic activation to cause rapid ROS generation. Thus, in order to prevent this occurrence, higher concentrations than have previously been studied are needed (Figure 7). While the authors note that some lower concentrations of BP nanoflakes exfoliated directly onto titanium substrates (900 ng cm^−2^) showed antimicrobial efficacy [5], these pure BP coatings relying on direct physical contact would mostly begin to react and decay upon exposure to air or humidity prior to implantation, thus limiting their use case.

To provide adequate antimicrobial activity from BP nanoflakes, some photoactive stimulation methods will be required, thus limiting its usage to topical antimicrobial agents or small implants. For larger orthopedic implants, BP-loaded hydrogels could make use of the osteogenic effects of BP [32]; however, utilizing the widespread antimicrobial effect may be challenging in surgical theaters.

## 3. Conclusions

BP nanoflakes in 20% *w*/*v* F127 proved to be ineffective as an antibacterial strategy without the use of NIR irradiation or ambient light conditions. At concentrations > 5120 μg/mL, bacterial inhibition was recorded; however, by counting colony-forming units, it was determined that it was not potent enough to kill all bacteria and prevent infections. For sub-lethal concentrations of <1280 μg/mL, optical density readings suggested an increase in bacteria activity, which may be attributed to the low ROS that may stimulate bacteria growth. Using small volumes of 50 μL, 20% F127 underwent rapid dissolution within 2 days, which should have exposed BP nanoflakes to the bacteria; however, without NIR or ambient light irradiation, the passive diffusion of black phosphorus did not provide adequate bactericidal activity. In situations where administering NIR or light is impractical, for instance, as an implant coating, conventional antibiotics are a more suitable option for infection prevention. The bactericidal activity of BP on other bacteria strains, including Gram-negative *E. coli*, could also be assessed; however, most implant infections are caused by *S. aureus,* which is why it was prioritized in this study. BP nanoflakes as an antimicrobial agent may be more suited to topical infections where light can easily be administered.

## 4. Experimental Section

### 4.1. Black Phosphorus Nanoflake Exfoliation

To process bulk black phosphorus powder (Ossila Limited, Sheffield, UK) into 2D nanoflakes, the liquid-phase exfoliation method was used. Bulk black phosphorus powder was suspended in 5 mL MilliQ water inside 20 mL scintillation vials (Figure 2). The suspension was then sonicated using an ultrasonic probe sonicator (Scientz, Ningbo, China) at 150 W in an ice bath for 6 h. This transferred shear forces to the bulk black phosphorus powder, exfoliating nanoflakes. Immediately after sonication, the BP solution was thoroughly mixed to evenly disperse the nanoflakes and was pipetted into separate vials according to the range of concentrations tested. Samples were then freeze-dried (Alpha 1-2 LDplus, Scitek, Australia) and stored in a dark environment to prevent oxidization and irradiation during storage.

### 4.2. BP F127 Hydrogel Preparation

In this study, 20% *w*/*v* Pluronic F127 hydrogels were prepared using the cold technique detailed by Schmolka I.R with sterile MilliQ water [33]. To prepare BP F127 hydrogels, 20% *w*/*v* F127 hydrogel was pipetted into the vials containing the freeze-dried BP nanoflakes, and the solution was vortexed. For antibacterial experiments, BP F127 gels were prepared on the same day and stored in the refrigerator at 4 °C to minimize the oxidation of BP before bacteria were exposed to the gels. Concentrations of 10,240, 5120, 2560, 1280, 640, 320, 160, and 80 µg/mL BP F127 were prepared by adding F127 to the pre-measured freeze-dried BP nanoflakes.

### 4.3. Black Phosphorus Nanoflake Characterization

SEM micrographs of the BP nanoflakes and freeze-dried BP F127 hydrogels were acquired using an FEI Quanta 200 ESEM (FEI, Hillsboro, OR, USA). SEM samples were prepared by pipetting a drop of BP nanoflake solution onto a silicon wafer, and they were dried on a hot plate. The samples were sputter-coated with 5 nm thick iridium film using an EM ACE600 sputter coater (Leica, Wetzlar, Germany). Micrographs were acquired using an acceleration voltage of 20 kV with a spot size of 5.

To image BP F127 hydrogels, samples were freeze-dried, crushed into a powder, mounted on carbon tape, and sputter-coated with 5 nm thick iridium film. For hydrogel samples, an acceleration voltage of 10 kV was used with a spot size of 4. EDS samples of BP F127 were prepared similarly but sputter-coated with 5 nm of carbon. The EDS map and spectra were acquired using an Oxford EDS (Oxford Instruments, Abingdon, Oxfordshire, UK) detector with the same acceleration voltage and spot size.

To prepare TEM samples, BP nanoflakes suspended in water were pipetted onto holey carbon TEM grids and left to dry. TEM micrographs were acquired using a JEOL 1010 microscope (JEOL, Tokyo, Japan) and operated at an acceleration voltage of 100 kV. Images were processed using DigitalMicrograph 3.50 and analyzed in Fiji (NIH, Bethesda, MD, USA).

To determine the approximate range of nanoflake sizes after 4 h of sonication in water, BP nanoflakes were examined by dynamic light scattering using ALV-5000 fast DLS (ALV, Langen/Hessen, Germany). The temperature was regulated at 20 °C by a water bath, and the scanning angle was 90°.

Raman spectroscopy was used to identify the presence of BP nanoflakes in all concentrations assessed. After dispensing the BP nanoflakes into separate vials according to the desired concentrations, 10 µL drops from each concentration were pipetted into a silicon substrate and dried on a hot plate. Raman spectra were acquired using a 532 nm wavelength laser with 1200 lines/mm grating on a WiTEC Raman system (Oxford Instruments, Abingdon, Oxfordshire, UK).

### 4.4. Pluronic F127 Dissolution Profile

In the BP F127 antibacterial experiment protocol, 50 µL of 20% *w*/*v* F127 was exposed to 200 µL of inoculated TSB medium at 37 °C. To determine if BP nanoflakes would degrade in the presence of bacteria or whether they would be self-contained within the F127 gel, an F127 dissolution experiment was conducted based on an established protocol from Diniz et al. [34]. In total, 50 µL of 20% Pluronic F127 was incubated in 1.5 mL microcentrifuge tubes until gelation. In total, 200 µL of 1× PBS was then added to the top of the gelated F127 and incubated at 37 °C for various time intervals (0 min-2 days). At each time interval, samples were centrifuged at 10,000× *g* for 5 min, and the solution was removed via micropipetting, leaving gelated F127 at the bottom of the vial. Samples were then air-dried for 48 h and weighed. The dissolution profile was evaluated by measuring the weight lost at each time interval.

### 4.5. Black Phosphorus Nanoflake F127 ROS Detection

The production of oxidative species through the breakdown of BP was assessed using various dyes using similar protocols from a prior study [35]. Specifically, xanthine oxidase (XO) was employed to detect superoxide radicals (O_2_^•−^); horse radish peroxidase (HRP) was used to detect the hydroxide radicals (OH^•^); and singlet oxygen (^1^O_2_) was detected using methylene blue (MB). In total, 5 mg HRP (Sigma) was added to 50 mL of Milli-Q water solution to make up HRP solution; 5 μM XO (Sigma) was mixed in 50 mL of Milli-Q water to prepare the xanthine oxidase solution; and 100 mg of methylene blue(sigma) was added into 50 mL of Milli-Q water to make up the MB solution. Equal concentrations of BP hydrogels (100 µL) and PBS (500 µL) were added to a 24-well plate prior to testing and incubated for 24 and 48 h at 37 °C. At each time point, the dye was added to each well and incubated for an additional 2 h. Post incubation, analysis was performed using a UV spectrophotometer (CLARIOstar) across the 220–800 nm wavelength. The absorbance peaks for ^1^O_2_, OH^•^, and O_2_^•−^ were observed at 650–700 nm, 350–450 nm, and 250–350 nm, respectively.

### 4.6. Optical Density of Staphylococcus Aureus at 600 nm

For the bacteria culture, *S. aureus* ATCC 25923 was streaked on tryptone soy agar (TSA) plates and incubated overnight. A single colony was selected using a sterile disposable loop and dispersed in 10 mL tryptone soya broth (TSB) in a 15 mL centrifuge tube. The centrifuge tube was placed in a shaking incubator overnight at 37 °C, with shaking at 200 rpm. After 16 h, the *S. aureus* liquid culture was diluted to an OD600 absorbance of 0.1 (10^7^ CFU/mL).

In a clear, flat-bottom 96 well plate (Thermo Scientific, Waltham, MA, USA), 50 µL of BP and VAN control F127 gels were micropipetted into the wells, and 50 µL of MilliQ water was used as a negative control. As the BP nanoflakes caused some absorption at OD600 that interfered with the results, the controls for each BP F127 concentration were prepared by pipetting 200 µL of sterile TSB media on top of the BP F127 gels to create a background (*n* = 4). For the inoculated experiment samples, the *S. aureus* liquid culture with an OD600 of 0.1 was further diluted 100 times in TSB media to 10^5^ CFU/mL, and 200 µL of the diluted liquid culture was pipetted into the wells and cultured in an incubator overnight at 37 °C (*n* = 4). After 24 h, 100 µL of TSB media from each well was transferred to another 96 well plate, and OD600 was recorded by a microplate reader (FLUOstar Omega, BMG Labtech, Ortenberg, Germany). The experiment results were subtracted by the respective mean negative control samples for each BP F127 concentration.

### 4.7. Colony-Forming Units of Staphylococcus Aureus Suspension

The bacterial solutions from each well from the previous step were subjected to a series of 10-fold dilutions in TSB. In total, 5 10 µL droplets from each dilution (10^−2^–10^−9^) were transferred onto a TSA plate. Following 20 h of incubation at 37 °C, the TSA plates were collected, and colonies were manually counted using ImageJ, version 1.54p (NIH, Bethesda, MD, USA).

### 4.8. Black Phosphorus Nanoflake F127 Disk Diffusion Test

Mueller–Hinton (MH) agar plates were thoroughly swabbed with the *S. Aureus* liquid culture with an OD600 absorbance of 0.1 to create an even lawn of bacteria. In total, 3 10 µL droplets of each F127 gel were transferred onto the lawn of bacteria. For the VAN control, three 6 mm circles of filter paper were placed on the surface of the MH agar, and 10 µL of 500 µg/mL VAN in Milli-Q was loaded onto the paper. Inoculated agar plates were incubated for 24 h at 37 °C, after which the zone of inhibition was measured with a ruler.

## Data Availability

Data is contained within the article or Supplementary Material.

## Supplementary Materials

The following supporting information can be downloaded at https://www.mdpi.com/article/10.3390/molecules30112292/s1, Figure S1. Dynamic Light Scattering distribution profile of exfoliated nanoflakes, sizes ranging from 679 ± 230 nm in width; Figure S2. EDS Spectra of BP F127 (2560 µg mL^−1^), sputter coated with carbon thread; Figure S3. Dissolution profile of 20% *w/v* F127 gel incubated in PBS at 37 °C over 2 days; Figure S4. ROS generation from BP F127; Table S1. Antimicrobial performance of BP nanoflakes. References from Table S1 are: [36,37,38,39,40,41,42,43,44,45,46,47,48,49,50].
