# Supplementary material for: Are Black Phosphorus Hydrogels Antimicrobial Without Photonic Activation?
Source: Molecules. 2025 May 23;30(11):2292. doi: 10.3390/molecules30112292 (PMC12155703; doi:10.3390/molecules30112292)
Supplement: Supplementary file 1 [file molecules-30-02292-s001.zip › molecules-3643242-supplementary.pdf]

Supporting Information

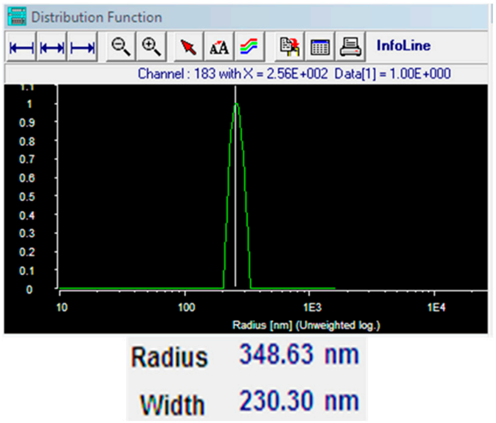

Figure S1. Dynamic Light Scattering distribution profile of exfoliated nanoflakes, sizes ranging from 679 ± 230 nm in width.

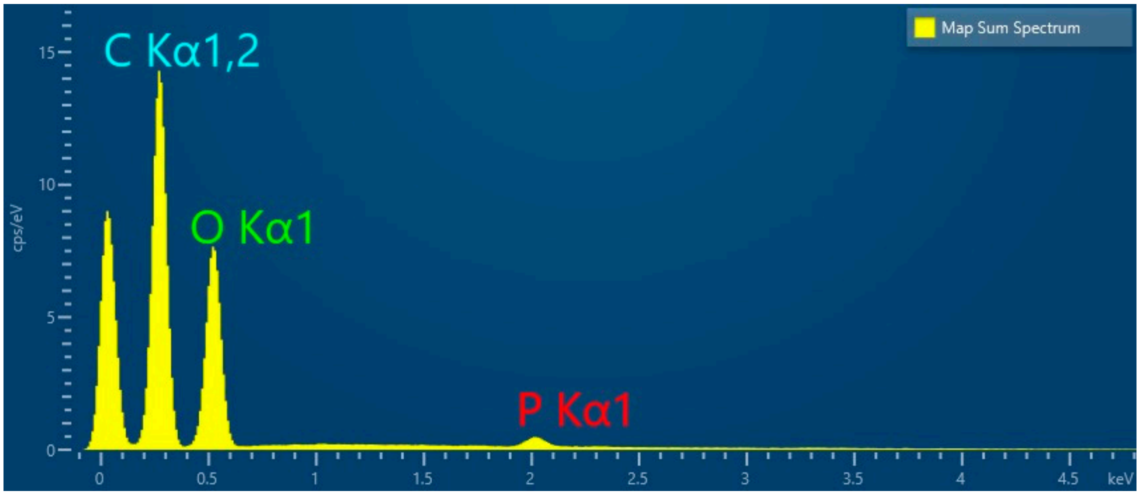

Figure S2. EDS Spectra of BP F127 (2560 µg mL-1), sputter coated with carbon thread. Phosphorus signal from BP nanoflakes are present when scanning the lyophilized hydrogel interface.

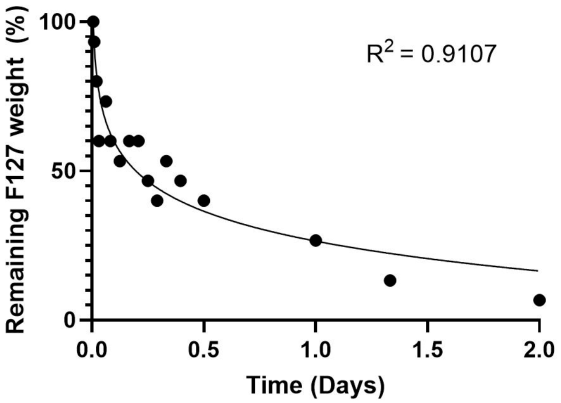

Figure S3. Dissolution profile of 20% w/v F127 gel incubated in PBS at 37⁰C over 2 days. The dissolution of F127 was fit to a logarithmic function.

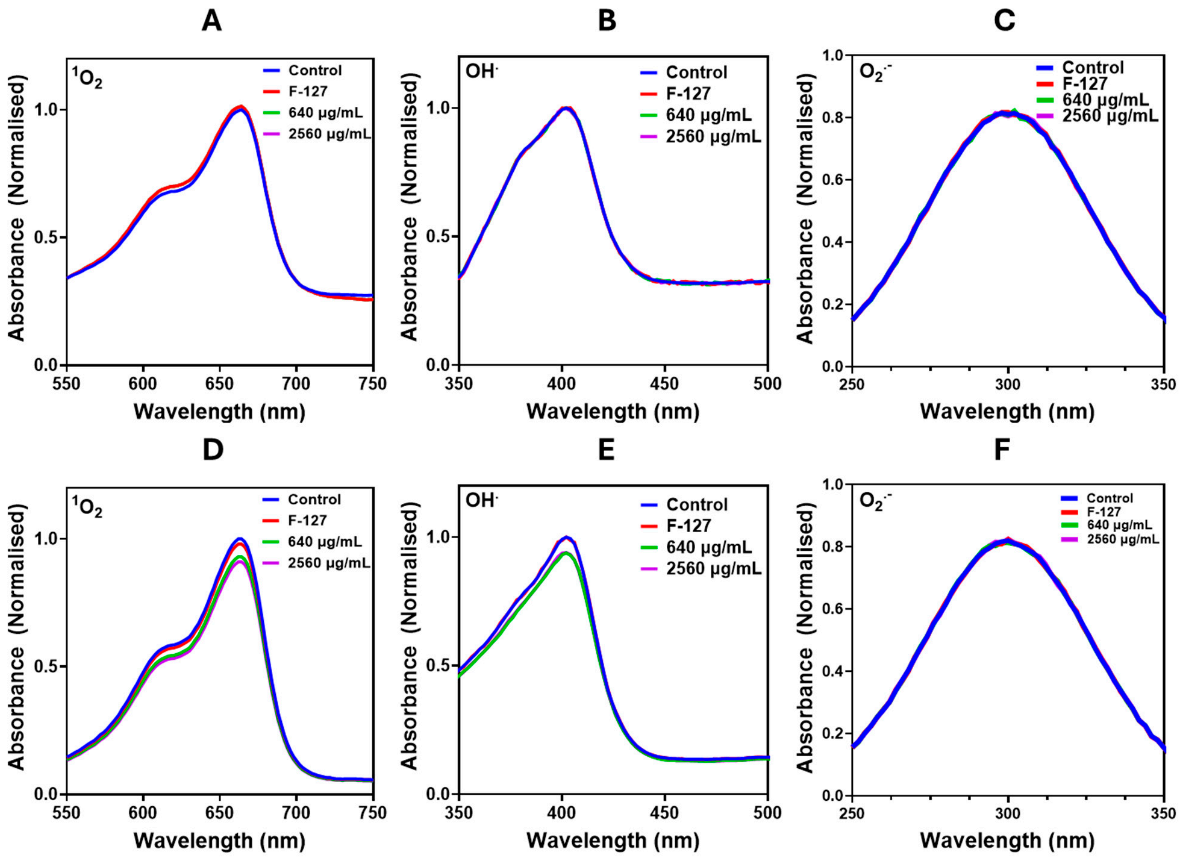

Figure S4. ROS generation. The UV absorbance spectra for $^1O_2$ (A), OH$^\bullet$ (B) and $O_2{\bullet}^-$ (C) radicals after 24 hours of incubation. The UV absorbance spectra for $^1O_2$ (D), OH$^\bullet$ (E) and $O_2{\bullet}^-$ (F) radicals after 48 hours of incubation.

Table S1. Antimicrobial performance of BP nanoflakes.

Ref	Delivery Method	BP Nanoflake Conc.	Bacteria	Bactericidal Performance	Suggested Antibacterial Mechanism
[1]	Exfoliated onto various substrates<br>(No hydrogel)	~900 ng cm−2	E. coli,<br>P. aeruginosa,<br>MRSA,<br>S. typhimurium,<br>B. cereus,	97.9 % MRSA and 99.3% for C. neoformans after 2 hours.<br><br>After 48 hours 63.9% ± 12.7% for MRSA and 73.3% ± 13.5% for C. neoformans	ROS generation. (No photonic activation)
[2]	Sterile water<br>(No hydrogel)	0.08-1.28 mg/mL	E. coli<br>S. aureus		Photothermal,<br>808 nm NIR laser, 1.0 W cm−2 for 3 min
[3]	GelMA-Dopamine methacrylate hydrogel	1 mg/mL	E. coli (ATCC25922) and S. aureus (CMCC26033)	85% for E. coli,<br>70% for S. aureus	Photothermal,<br>808 nm NIR laser for 10 min (1Wcm−2)
[4]	GelMA hydrogel	0.15-0.3 mg/mL	E. coli<br>S. aureus	94.53 ± 1.11% for E. coli<br><br>92.71 ± 1.68% for S. aureus<br><br>(For GelMA-BP, not BP+Mg gels)	Photothermal,<br>808 nm NIR laser for 5 min (1Wcm−2)
[5]	GelMA, Dextran, BP and Zinc nanoparticles hydrogel	0.1 mg/mL	E. coli<br>S. aureus	~80% for both bacteria using	Photothermal,

				GelMa+BP, ~95% for GelMA+BP+Zn	808 nm NIR laser (1.5 W/cm2) for 5 min
[6]	Chitosan/hydroxypropyltrimethyl ammonium chloride chitosan/hydroxyapatite/black phosphorus (CS/HC/HA/BP) scaffold.<br>(No hydrogel)	"75, 150, and 300 ppm "	E. coli<br>S. aureus	*E. coli* (97.38 ± 1.8 %) and *S. aureus* (92.2 ± 2.5 %)	Photothermal,<br>808 nm NIR laser for 10 min (1.5W cm−2)
[7]	Chitosan-based hydrogel	1mg/mL	E. coli,<br>S. aureus	Almost all bacteria (measured via colony counting)	Photothermal,<br>808 nm NIR laser for 10 min (1Wcm−2)
[8]	Hyaluronic acid hydrogel	0.2mg/mL	E. coli,<br>S. aureus	>99.99% for both bacteria	Photothermal,<br>808 nm NIR laser for 10 min (1Wcm−2)
[9]	Hyaluronic-acid hydrogel with silver nanoparticles	0.2 mg/mL	S. aureus	Not quantified as percentage	Photothermal,<br>808 nm NIR laser for 10 min (1Wcm−2)
[10]	Dopamine-Hyaluronic acid-F127 hydrogel	2mg/mL	E. coli<br>S. aureus	~2.2 log10 CFU for E. coli<br><br>~4 log 10 CFU for S. aureus	Photothermal,<br>808 nm NIR laser for 5 min (1Wcm−2)
[11]	Sterile water injection.<br><br>(No hydrogel)	1-1.500 mg/mL	S. aureus,<br>MRSA,<br>S. epidermidis,<br>P. aeruginosa,<br>E. coli	99% after 24 hours for S. aureus.<br>80% for P. aeruginosa	ROS generation stimulated by ambient light
[12]	Sterile water<br>(No hydrogel)	0.1 mg/mL	E. coli<br>B. subtilis	91.65 ± 2.14% for E. coli after 12 hours	ROS generation. (No photonic activation)

				99.99 ± 0.01% for B. subtilis after 6 hours	
[13]	Eutectogels (Choline chloride:glycerol-cellulose)	2.5 mg/g	MRSA, P. aeruginosa, C. albicans	>90% reduction in all microbials	ROS generation stimulated by ambient light
[14]	Hydrogel -Dopamine-functionalized oxidized hyaluronic acid (OHA-DA) and cyanoacetategroup-functionalized dextran (DEX-CA)	0.5 mg/mL	S. aureus, E. coli	96.99% 96.2%	Photothermal, 808nm NIR laser irradiation (1Wcm−2)
[15]	3-aminophenylboronic acid modified oxidized chondroitin sulfate, polyvinyl alcohol (PVA), black phosphorus/bismuth oxide/ε-polylysine hydrogel	0.2 mg/mL	E. coli, S. aureus P. aeruginosa	84–89 % bactericidal	Photothermal, 808 nm NIR laser for 10 min (1Wcm−2)
[16]	poly (lactic-co-glycolic) acid (PLGA), Chitosan, Gelatin<br><br>(No hydrogel)	0.15, 0.3, 0.5 mg/mL	E. coli<br>S. aureus<br>P. aeruginosa	~95% for 0.5 mg/mL	Photothermal, 808 nm NIR laser (1.5 W/cm2) for 5 min
[17]	Polydopamine-based Titanium surface coating with BP and Zn nanowires<br><br>(No hydrogel)	20mg/mL solution, shaken with dopamine to coat implants	E. coli<br>S. aureus	~55% for Ti-PDA/BP. 99.3% with Ti-PDA/BP/ZnO	Photothermal, 808 nm NIR irradiation for 5 min (0.5 W cm^{-2})
[18]	poly(vinyl alcohol)/chitosan hydrogel with BP and magnesium oxide nanoparticles hydrogel	50 ppm	E. coli<br>S. aureus	99.4% bactericidal with NIR	Photothermal, 808 nm NIR laser (1.0 W/cm2) for 5 min
[19]	GelMA, 4-octyl itaconate-modified BP hydrogel	0.05 mg/mL	E. coli<br>S. aureus	92.27% for E. coli and 96.69% for S. aureus	Photothermal, 808 nm NIR laser (1.0 W/cm2) for 5 min

[20]	PVA and sodium alginate hydrogel with BP quantum dots	0.05-0.2 mg/mL	E. coli<br>S. aureus<br>MRSA	95% for MRSA	Phototherm al,<br>808 nm NIR laser<br>(1.0 W/cm2) for 4 min
[21]	Hydroxyapatite- based “biopaper” with BP nanosheets<br>(No hydrogel)	2% w/v BP to biopaper	E coli,<br>S. aureus	97.0% for S. aureus	Phototherm al,<br>808 nm NIR laser<br>(1.0 W/cm2) for 10 min
[22]	BP- Chitosan implant coating on 3D printed PEEK polymer<br><br>(No hydrogel)	-	E coli,<br>S. aureus	E. coli (91.07 ± 6.00%)<br>S. aureus (85.61 ± 6.04%)	Phototherm al,<br>808 nm NIR laser<br>(0.77 W/cm^2) for 10 min
[23]	silk fibroin,<br>gelatin, agarose, and black phosphorus quantum dots (BPQDs)<br>(No hydrogel)	0.05-0.2 mg/mL	E. coli	99%	Phototherm al,<br>808 nm NIR laser<br>(2.41 W) for 10 min
[24]	Polyethyleneimine-based hydrogel with BP quantum dots	0.05 mg/mL	E. coli,<br>S. aureus,<br>P. aeruginosa,	99%	Phototherm al,<br>808 nm laser (0.5 W cm−2) for 10 min
[25]	GelMA	0.04-0.2% w/v	S. aureus	98%	Phototherm al,<br>808 nm NIR laser<br>(1.0 W/cm2) for 5 min
[26]	poly(d,l-lactide)-poly(ethylene glycol)-poly(d,l-lactide) hydrogel	2-50 ppm	S. aureus	99.5%	Phototherm al,<br>808 nm, 1.0 W cm−2 for 5 min
[27]	Chitosan hydrogel	0.2 mg/mL	E. coli<br>S. aureus	95.6% for E. coli<br><br>94.56% for S. aureus	Phototherm al,<br>300 W xenon lamp for 10 minutes

[1] Z. L. Shaw, S. Kuriakose, S. Cheeseman, E. L. H. Mayes, A. Murali, Z. Y. Oo, T. Ahmed, N. Tran, K. Boyce, J. Chapman, C. F. McConville, R. J. Crawford, P. D.

Taylor, A. J. Christofferson, V. K. Truong, M. J. S. Spencer, A. Elbourne, S. Walia, Broad-Spectrum Solvent-free Layered Black Phosphorus as a Rapid Action Antimicrobial. ACS Appl Mater Interfaces 13, 17340–17352 (2021).

[2] Z. Sun, Y. Zhang, H. Yu, C. Yan, Y. Liu, S. Hong, H. Tao, A. W. Robertson, Z. Wang, A. A. H. Pádua, New solvent-stabilized few-layer black phosphorus for antibacterial applications. Nanoscale 10, 12543–12553 (2018).

[3] Y. Li, C. Liu, X. Cheng, J. Wang, Y. Pan, C. Liu, S. Zhang, X. Jian, PDA-BPs integrated mussel-inspired multifunctional hydrogel coating on PPENK implants for anti-tumor therapy, antibacterial infection and bone regeneration. Bioact Mater **27**, 546–559 (2023).

[4] X. R. Jing, C. Xu, W. J. Su, Q. Y. Ding, B. Ye, Y. L. Su, K. D. Yu, L. Zeng, X. Yang, Y. Z. Qu, K. F. Chen, T. F. Sun, Z. Q. Luo, X. D. Guo, Photosensitive and Conductive Hydrogel Induced Innerved Bone Regeneration for Infected Bone Defect Repair. Adv Healthc Mater **12** (2023).

[5] L. Zhou, L. Zhou, C. Wei, R. Guo, A bioactive dextran-based hydrogel promote the healing of infected wounds via antibacterial and immunomodulatory. Carbohydr Polym **291**, 119558 (2022).

[6] Y. Zhao, X. Peng, X. Y. Xu, M. Z. Wu, F. Sun, Q. W. Xin, H. B. Zhang, L. R. Zuo, Y. L. Cao, Y. H. Xia, J. Luo, C. M. Ding, J. S. Li, Chitosan based photothermal scaffold fighting against bone tumor-related complications: Recurrence, infection, and defects. Carbohydr Polym **300** (2023).

[7] J. Zhou, T. Li, M. Zhang, B. Han, T. Xia, S. Ni, Z. Liu, Z. Chen, X. Tian, Thermosensitive black phosphorus hydrogel loaded with silver sulfadiazine promotes skin wound healing. J Nanobiotechnology **21**, 330 (2023).

[8] X. Yang, S. He, J. Wang, Y. Liu, W. Ma, C.-Y. Yu, H. Wei, Hyaluronic acid-based injectable nanocomposite hydrogels with photo-thermal antibacterial properties for infected chronic diabetic wound healing. Int J Biol Macromol **242**, 124872 (2023).

[9] Y. C. Zhao, Z. J. Chen, W. J. Shao, S. Yang, W. G. Cui, Z. W. Cai, L. Cheng, R. X. Lin, Black phosphorus-enhanced injectable hydrogel for infected soft tissue healing. APL Bioeng **7** (2023).

[10] Z. M. Li, L. Yang, D. Zhang, W. Y. Wang, Q. L. Huang, Q. Y. Liu, K. X. Shi, Y. K. Yu, N. S. Gao, H. Z. Chen, S. Y. Jiang, Z. J. Xie, X. W. Zeng, Mussel-inspired "plug-and-play" hydrogel glue for postoperative tumor recurrence and wound infection inhibition. J Colloid Interface Sci **650**, 1907–1917 (2023).

[11] E. P. Virgo, H. Haidari, Z. L. Shaw, L. Z. Y. Huang, T. L. Kennewell, L. Smith, T. Ahmed, S. J. Bryant, G. S. Howarth, S. Walia, A. J. Cowin, A. Elbourne, Z. Kopecki, Layered Black Phosphorus Nanoflakes Reduce Bacterial Burden and Enhance Healing of Murine Infected Wounds. Adv Ther (Weinh) **6**, 2300235 (2023).

[12] Z. Xiong, X. Zhang, S. Zhang, L. Lei, W. Ma, D. Li, W. Wang, Q. Zhao, B. Xing, Bacterial toxicity of exfoliated black phosphorus nanosheets. Ecotoxicol Environ Saf 161, 507–514 (2018).

[13] Z. L. Shaw, M. N. Awad, S. Gharehgozlo, T. L. Greaves, H. Haidari, Z. Kopecki, G. Bryant, P. T. Spicer, S. Walia, A. Elbourne, S. J. Bryant, Deep Eutectic Solvent

Eutectogels for Delivery of Broad-Spectrum Antimicrobials. ACS Appl Bio Mater, doi: 10.1021/acsabm.3c00971 (2023).

[14] X. Y. Ding, Y. R. Yu, L. Fan, W. Z. Li, F. K. Bian, J. L. Wang, Y. J. Zhao, Sprayable Multifunctional Black Phosphorus Hydrogel with On-Demand Removability for Joint Skin Wound Healing. Adv Healthc Mater **13** (2024).

[15] Y. Zhang, W. J. Chen, W. J. Feng, W. H. Fang, X. Han, C. Cheng, Multifunctional chondroitin sulfate based hydrogels for promoting infected diabetic wounds healing by chemo-photothermal antibacterial and cytokine modulation. Carbohydr Polym **314** (2023).

[16] L. Zhou, N. Liu, L. Feng, M. Zhao, P. Wu, Y. Chai, J. Liu, P. Zhu, R. Guo, Multifunctional electrospun asymmetric wettable membrane containing black phosphorus/Rg1 for enhancing infected wound healing. Bioeng Transl Med **7** (2022).

[17] J. Fang, Y. Wan, Y. Sun, X. Sun, M. Qi, S. Cheng, C. Li, Y. Zhou, L. Xu, B. Dong, L. Wang, Near-infrared-activated nanohybrid coating with black phosphorus/zinc oxide for efficient biofilm eradication against implant-associated infections. Chemical Engineering Journal **435**, 134935 (2022).

[18] Qing, Y. A.; Wang, H.; Lou, Y.; Fang, X.; Li, S. H.; Wang, X. Y.; Gao, X.; Qin, Y. G. Chemotactic Ion-Releasing Hydrogel for Synergistic Antibacterial and Bone Regeneration. *Mater Today Chem* **2022**, *24*. https://doi.org/10.1016/j.mtchem.2022.100894.

[19] Q. Y. Ding, T. F. Sun, W. J. Su, X. R. Jing, B. Ye, Y. L. Su, L. Zeng, Y. Z. Qu, X. Yang, Y. Z. Wu, Z. Q. Luo, X. D. Guo, Bioinspired Multifunctional Black Phosphorus Hydrogel with Antibacterial and Antioxidant Properties: A Stepwise Countermeasure for Diabetic Skin Wound Healing. Adv Healthc Mater **11** (2022).

[20] S. C. Huang, S. B. Xu, Y. A. Hu, X. J. Zhao, L. N. Chang, Z. H. Chen, X. F. Mei, Preparation of NIR-responsive, ROS-generating and antibacterial black phosphorus quantum dots for promoting the MRSA-infected wound healing in diabetic rats. Acta Biomater 137, 199–217 (2022).

[21] J. K. Zeng, X. W. Geng, Y. F. Tang, Z. C. Xiong, Y. J. Zhu, X. S. Chen, Flexible photothermal biopaper comprising Cu2+-doped ultralong hydroxyapatite nanowires and black phosphorus nanosheets for accelerated healing of infected wound. CHEMICAL ENGINEERING JOURNAL **437** (2022).

[22] He, M. M.; Zhu, C.; Sun, D.; Liu, Z.; Du, M. X.; Huang, Y.; Huang, L. Z.; Wang, J. H.; Liu, L. M.; Li, Y. B.; Song, Y. M.; Feng, G. J.; Zhang, L. Layer-by-Layer Assembled Black Phosphorus/Chitosan Composite Coating for Multi-Functional PEEK Bone Scaffold. *COMPOSITES PART B-ENGINEERING* **2022**, *246*.

[23] H. Zhang, Z. H. Zhang, H. Zhang, C. W. Chen, D. G. Zhang, Y. J. Zhao, Protein-Based Hybrid Responsive Microparticles for Wound Healing. ACS Appl Mater Interfaces **13**, 18413–18422 (2021).

[24] L. P. Zhou, W. Pi, S. Y. Cheng, Z. Gu, K. X. Zhang, T. T. Min, W. M. Zhang, H. W. Du, P. X. Zhang, Y. Q. Wen, Multifunctional DNA Hydrogels with Hydrocolloid-Cotton Structure for Regeneration of Diabetic Infectious Wounds. Adv Funct Mater 31 (2021).

[25] Y. Miao, X. Shi, Q. Li, L. Hao, L. Liu, X. Liu, Y. Chen, Y. Wang, Engineering natural matrices with black phosphorus nanosheets to generate multi-functional therapeutic nanocomposite hydrogels †. Cite this: Biomater. Sci 7, 4046 (2019).
[26] J. Shao, C. Ruan, H. Xie, Z. Li, H. Wang, P. K. Chu, X. F. Yu, Black-Phosphorus-Incorporated Hydrogel as a Sprayable and Biodegradable Photothermal Platform for Postsurgical Treatment of Cancer. Advanced Science 5 (2018).
[27] C. Y. Mao, Y. M. Xiang, X. M. Liu, Z. D. Cui, X. J. Yang, Z. Y. Li, S. L. Zhu, Y. F. Zheng, K. W. K. Yeung, S. L. Wu, Repeatable Photodynamic Therapy with Triggered Signaling Pathways of Fibroblast Cell Proliferation and Differentiation To Promote Bacteria-Accompanied Wound Healing. ACS Nano 12, 1747–1759 (2018).
